# Preoperative versus postoperative initiation of thromboprophylaxis following major orthopedic surgery: safety and efficacy of postoperative administration supported by recent trials of new oral anticoagulants

**DOI:** 10.1186/1477-9560-9-17

**Published:** 2011-11-16

**Authors:** Carsten Perka

**Affiliations:** 1 Orthopaedic Department, Charité, University Medicine Berlin, Free and Humboldt-University of Berlin, Berlin, Germany

**Keywords:** Thromboprophylaxis, Hip replacement surgery, Knee replacement surgery, Anticoagulation, Dabigatran etexilate, Rivaroxaban, Apixaban

## Abstract

In European countries, low-molecular-weight heparin is generally initiated preoperatively for thromboprophylaxis in hip or knee replacement surgery. The objective of this review is to compare pre- and postoperative thromboprophylaxis strategies using available evidence, and discuss the challenges and issues that arise. Surgery is the first step in the process of thrombus formation, but thrombosis is not an instant process and the formation and growth of the thrombus can take several days or weeks. Hence, it may be possible to stop this process if thromboprophylaxis is initiated after surgery. Meta-analyses or systematic reviews comparing pre- and postoperative initiation of therapy have found no consistent differences in efficacy and similar safety (bleeding rates) between the two strategies. The recently available oral anticoagulant dabigatran etexilate provides thromboprophylaxis when administered postoperatively and is as safe as preoperative enoxaparin. Further support for the use of postoperative oral thromboprophylaxis in hip or knee replacement surgery has been provided by the phase III clinical trials of rivaroxaban and apixaban versus preoperative enoxaparin. Postoperative thromboprophylaxis offers the opportunity to change management practices in Europe. As postoperative initiation may have a clinical benefit in some settings (e.g. for neuraxial anesthesia) and practical advantages (e.g. allowing same-day admission), it is a worthwhile thromboprophylactic strategy for hip or knee replacement surgery.

## Introduction

Venous thromboembolism (VTE) is a serious complication of elective hip and knee replacement surgery. Without thromboprophylaxis, VTE occurs in approximately 40-60% of cases. Hence, evidence-based guidelines recommend thromboprophylaxis for all patients undergoing hip or knee replacement surgery [1,2].

In many European countries, low-molecular-weight heparin (LMWH) is considered the standard therapy for prophylaxis following hip or knee replacement surgery and is initiated preoperatively to maximize efficacy [3]. Preoperative thromboprophylaxis is initiated on the assumption that the surgery itself and the accompanying immobility are the main causes of thrombosis [4-7]. However, as most thrombi develop postoperatively, starting anticoagulant therapy following surgery could also prevent VTE [8-10].

Initiation of thromboprophylaxis after surgery has several potential advantages. It simplifies same-day admission for elective procedures and, as therapy is initiated after surgery when patients are hemodynamically stable, there is a lower risk of bleeding. Neuraxial anesthesia is increasingly used in orthopedic surgery, but there is a risk of spinal hematoma and subsequent paralysis, which could be increased by the sustained use of an anticoagulant [11]. Initiation of thromboprophylaxis with LMWH too close to the start of surgery (from -2 h to +4 h of surgery) is associated with an increased risk of bleeding and neuraxial compressive hematoma, so delaying thromboprophylaxis until after a stable clot has been established at the injection site would seem sensible [5,12,13]. The timing of dosing would be dependent upon the onset time (*t*_max_) of the anticoagulant used [13]. In acknowledgement of the increased risk of hematoma associated with preoperative thromboprophylaxis, recent guidelines from the American Society of Regional Anesthesia and Pain Medicine recommend that needle placement should occur at least 10 to 12 h after a preoperative LMWH dose so that the procedure it not carried out during peak anticoagulant activity [14]. With postoperative initiation of thromboprophylaxis, therapy is not initiated until several hours after catheter withdrawal, which may help to improve the safety of neuraxial blockade [1].

A further aspect of preoperative thromboprophylaxis is that considerable care must be taken with patients with impaired renal function to ensure that they are not put at an increased risk of perioperative bleeding as a result of the reduced renal excretion of LMWH, subsequent increased half-life and potential drug accumulation [15-18].

The advent of oral anticoagulants that provide effective thromboprophylaxis when initiated postoperatively (e.g. dabigatran etexilate, rivaroxaban and apixaban) raises the question of whether European practice should be reconsidered [19-26]. Here, I review the information available based on phase III data for these three novel anticoagulation agents initiated postoperatively, in comparison with the standard European regimen of LMWH initiated preoperatively, and discuss the challenges and questions that arise.

### Pathophysiology of thrombus formation in orthopedic surgery

Hemostasis is a normal biological process involving the coagulation cascade. In essence, damage to a blood vessel wall initiates hemostasis, leading to activation of platelets and coagulation factors. Thrombin is central to this process and is produced on the surface of the activated platelets. An amplification system leads to additional platelet and clotting factor activation, and more thrombin production. Once produced, without thromboprophylaxis, thrombin converts fibrinogen to fibrin, which provides a structural network for the formation of the clot [27].

VTE occurs due to an imbalance in thrombin activity. For this to happen, three factors, known as Virchow's triad, must be present: vascular injury, alterations in blood flow, and activation of coagulation [28]. In addition, other independent risk factors for VTE may be present, such as patients being over 70 years of age, having concomitant medical conditions, and use of general anesthesia. The latter is implicated as a risk factor as it reduces blood flow to the lower limbs [29].

The risk of VTE following total knee or hip replacement surgery is especially high as several pro-thrombotic processes are involved: coagulation activation from tissue and bone injury; vein dilation or injury with endothelial damage; vein distortion during surgery; heat due to cement polymerization in total hip replacement; patient immobility causing venous stasis; and reduced venous emptying peri- or post-surgery [30,31]. The scale of this adverse consequence of hip and knee surgery is demonstrated by the fact that 50% and 40%, respectively, of all diagnosed deep vein thromboses (DVT) are located in the proximal leg veins [9]. While surgery may be the event that initiates thrombus formation, it is not an instant process. Formation and growth of the thrombus can take several days or weeks and requires prolonged thromboprophylaxis, as discussed in the next section.

### Timing of thrombus formation

Several studies have examined the occurrence of symptomatic thrombosis following orthopedic surgery and have concluded that, in general, symptomatic thrombosis presents after discharge from hospital and is the most common cause for hospital readmission after hip replacement [8,32,33]. The proportion of symptomatic VTE events occurring after discharge from hospital ranges from 35% to 76% depending on the study and the type of surgery [32,34]. The incidence of asymptomatic DVT, as demonstrated by venography, is much higher than that of symptomatic VTE following major orthopedic surgery [35]. A recent retrospective review of 12 studies undertaken in patients undergoing elective total hip or total knee replacement surgery investigated the relationship between asymptomatic DVT and the subsequent development of symptomatic VTE [36]. The 3-month incidence of asymptomatic DVT was 13.2% after total hip replacement and 38.1% after total knee replacement compared with rates of symptomatic VTE of 2.7% and 1.8%, respectively; i.e. one symptomatic VTE developed for every five asymptomatic DVTs after total hip replacement surgery compared with one symptomatic VTE for every 21 asymptomatic DVTs after total knee replacement surgery [35].

Regarding the timing of symptomatic VTE, Bjørnarå et al. report that most symptomatic cases of VTE following orthopedic surgery occur within 3 months of the operation, with a median time to occurrence of symptomatic DVT and pulmonary embolism of 21 and 34 days following hip replacement, and of 20 and 12 days, respectively, following knee replacement [8]. Similarly, Dahl et al. report development of symptomatic DVT, on average, 27 days after hip replacement and 16 days after knee replacement, while the RIETE Registry reports a mean time to clinically overt pulmonary embolism of 22 ± 16 days in patients undergoing major orthopedic surgery [9,10]. In addition, the risk of developing symptomatic VTE lasts for up to 3 months after hip replacement and up to 1 month after knee replacement [1,8]. Given the evidence suggesting that symptomatic VTE can develop up to 3 months following surgery and that there are large numbers of asymptomatic DVTs that could become symptomatic, thromboprophylactic treatment for up to 35 days post-surgery is recommended [1]. Several studies show that the onset of asymptomatic VTE can also occur several weeks after total hip replacement; about 20-30% of those who had no DVT detectable by venography at 7 or 10 days post-surgery (when prophylaxis was stopped) had evidence of asymptomatic DVT on their venograms at weeks 4 to 5 after surgery [13,36-39]. Furthermore, thromboprophylaxis has been shown to reduce the incidence of asymptomatic and symptomatic VTE, and a longer duration of prophylaxis provides greater protection than a shorter duration [40-42].

### Preoperative initiation of thromboprophylaxis

The initial trials of LMWH showed an increase in bleeding if the first dose of 5000 or 2500 U was given 2 h preoperatively [43]. However, subsequent European trials have demonstrated the safety and efficacy of LMWH for preventing VTE following hip and knee replacement surgery when initiated 12 h preoperatively [44]. Hence, the regimen in Europe is generally to administer LMWH once daily (qd), starting 12 h before surgery, which may reflect the European preference for single-daily dosing.

The rationale behind this approach is based on the assumption that the surgical procedure and associated immobility is the main initiator of thrombosis; administering prophylaxis before surgery could, therefore, allow optimal antithrombotic treatment [4-7]. However, as already discussed, the majority of thrombi are formed days, if not weeks, after surgery and would still be prevented if the first dose was delayed until after the operation [8-10]. In addition, initiating therapy 12 h before surgery means that much of the drug has been eliminated by the time of surgery. For example, the elimination half-life of enoxaparin sodium is ~4 h after a single subcutaneous (sc) dose and ~7 h after repeated dosing; significant anti-factor Xa activity persists in plasma for ~12 h following a 40-mg single sc dose, while the steady state is achieved on the second day of treatment [45]. This can be viewed as beneficial as it reduces the risk of intraoperative bleeding, but one could also argue that the antithrombotic effect is minimal and the majority of the protective effect comes from subsequent doses given after surgery. Thus, this calls into question the value of preoperative administration of prophylactic anticoagulants.

### Postoperative initiation of thromboprophylaxis

In the USA and Canada, more emphasis has traditionally been placed on the risk of bleeding than on efficacy when considering prevention of VTE [46]. Indeed, the 7th edition of the American College of Chest Physicians (ACCP) guidelines state: '...we place ... a relatively high value on minimizing bleeding complication' [47]. An influential trial of LMWH twice daily (bid) initiated postoperatively versus placebo was performed by Turpie et al. and showed effective thromboprophylaxis without excessive bleeding [48]. As a result, most subsequent US trials investigated postoperative initiation of thromboprophylaxis, thereby establishing its efficacy and safety [44]. Consequently, standard practice in North America is to administer therapy (30 mg enoxaparin bid) starting 12-24 h postoperatively once hemostasis has been established.

The timing of therapy initiation with this approach addresses concerns regarding bleeding, while use of a larger total daily dose recognizes that some thrombi may already have formed and that their growth may be slowed, enabling fibrinolysis [49]. The adoption of the bid regimen was further driven by the initial approval of LMWH given by the regulatory agencies, which was based on the half-life of LMWH [45]. The accumulated data from the US experience with LMWH support postoperative initiation of thromboprophylaxis as a safe, effective and convenient regimen.

### Preoperative initiation vs. postoperative initiation of thromboprophylaxis

The historical data suggest that both preoperative initiation and postoperative initiation of thromboprophylaxis are safe and effective regimens. Meta-analyses or systematic reviews comparing pre- and postoperative initiation of therapy have found no consistent difference in efficacy and safety (bleeding rates) between the two strategies [5,44,50]. However, the limitations common to all meta-analyses or systematic reviews and specific to these analyses (e.g. small sample sizes in some studies, differences in study conduct, etc.) mean that these studies can only provide an indication of relative efficacy and safety of the two strategies. Well-designed studies with large sample sizes directly comparing the two strategies provide more robust evidence. Data generated during the development of dabigatran etexilate, rivaroxaban and apixaban provide these kind of head-to-head data, and give an insight into the benefit: risk ratio of these novel anticoagulants initiated postoperatively compared with the European standard dose of enoxaparin started preoperatively.

Dabigatran etexilate was studied as thromboprophylaxis following elective total knee and hip replacement surgery in three European trials (Table 1) [19-21]. In all three studies, oral dabigatran etexilate was initiated as a half-dose 1-4 h post-surgery (as long as patients showed good hemostasis) and continued by using the full dose qd from the following day onwards. Reducing the first dose of dabigatran etexilate on the day of surgery with the full dose thereafter has been shown to improve the safety profile of the anticoagulant [51]. The comparator was 40 mg sc qd enoxaparin initiated 12 h before surgery. The end-point in the three studies was a composite of the incidence of total VTE and all-cause mortality, while the main safety outcome were the occurrence of bleeding events defined according to accepted guidelines [52]. Both doses of dabigatran etexilate tested (150 and 220 mg) had similar efficacy and safety to enoxaparin 40 mg (Table 1). Thus, as anticipated, bleeding rates were comparable between dabigatran etexilate and enoxaparin, while initiating dabigatran etexilate therapy post-surgery also effectively prevented or inhibited the process of clot formation.

**Table 1:** Efficacy and safety data from the three trials comparing dabigatran etexilate with the European enoxaparin dose as thromboprophylaxis following elective total hip or knee replacement surgery [19-21]

	220 mg dabigatran etexilate initiated post-surgery	150 mg dabigatran etexilate initiated post-surgery	40 mg enoxaparin initiated 12 h pre-surgery
**RE-MODEL™trial (knee replacement)**			
Total VTE and all-cause mortality (primary efficacy end-point)	183/503 (36.4%)	213/526 (40.5%)	193/512 (37.7%)
Major bleeding	10/679 (1.5%)	9/703 (1.3%)	9/694 (1.3%)
**RE-NOVATE^® ^trial (hip replacement)**			
Total VTE and all-cause mortality (primary efficacy end-point)	53/880 (6.0%)	75/874 (8.6%)	60/897 (6.7%)
Major bleeding	23/1146 (2.0%)	15/1163 (1.3%)	18/1154 (1.6%)
**RE-NOVATE^® ^II trial (hip replacement)**			
Total VTE and all-cause mortality (primary efficacy end-point)	61/792 (7.7%)	-	69/785 (8.8%)
Major bleeding	14/1010 (1.4%)	-	9/1003 (0.9%)

*VTE *venous thromboembolism

Support for the value of postoperative prophylaxis is also provided by studies comparing oral rivaroxaban 10 mg qd administered 6-8 h following surgery with enoxaparin 40 mg sc qd administered preoperatively (Table 2) [22-24]. It should be noted that rivaroxaban is administered a little later after wound closure than dabigatran etexilate (1-4 h). While postoperative initiation was effective, a major limitation to evaluating the comparative safety of rivaroxaban is the unique bleeding definition used in the studies [22-24]. Analyses of the complete rivaroxaban program with a more sensitive composite bleeding end-point (major bleeding plus clinically relevant non-major bleeding, as used in other trials) showed a significant higher bleeding rate for rivaroxaban compared with enoxaparin (3.2 vs. 2.5%; *P *= 0.039 [53]; 3.1 vs. 2.5%; *P *= 0.049) [53]. This is the expected profile of a relatively high-dose anticoagulant that provides greater efficacy compared with enoxaparin therapy at a cost of a greater risk of bleeding, and is a feature of the therapy rather than the timing of administration. However, in the same analysis, dabigatran etexilate showed no differences in bleeding rates compared with enoxaparin treatment, underlining the safety of this molecule [54].

**Table 2:** Efficacy and safety data from the three trials comparing rivaroxaban with the European enoxaparin dose as thromboprophylaxis following elective total hip or knee replacement surgery [22-24]

	10 mg rivaroxaban initiated post-surgery	40 mg enoxaparin initiated 12 h pre-surgery
**RECORD1 trial (hip replacement)**		
Total VTE and all-cause mortality (primary efficacy end-point)	18/1595 (1.1%)	58/1558 (3.7%)
Major bleeding	6/2209 (0.3%)	2/2224 (0.1%)
RECORD2 trial (hip replacement)*		
Total VTE and all-cause mortality (primary efficacy end-point)	17/864 (2.0%)	81/869 (9.3%)
Major bleeding	1/1228 (0.08%)	1/1229 (0.08%)
**RECORD3 trial (knee replacement)**		
Total VTE and all-cause mortality (primary efficacy end-point)	79/824 (9.6%)	166/878 (18.9%)
Major bleeding	7/1220 (0.6%)	6/1239 (0.5%)

*VTE *venous thromboembolism

*In RECORD2, rivaroxaban was given for 31 to 39 days, while enoxaparin was given for 10 to 14 days

Two phase III apixaban trials compared oral apixaban 2.5 mg bid started 12-24 h after orthopedic surgery with enoxaparin 40 mg sc qd administered 12 h preoperatively (Table 3) [25,26]. Both trials demonstrated that apixaban was more effective than the European enoxaparin regimen for the primary efficacy outcome and there was no significant difference in the rate of major or clinically relevant bleeding [25,26]. Thus, these results also support the use of postoperative rather than preoperative administration of thromboprophylactic agents after major orthopedic surgery.

**Table 3:** Efficacy and safety data from the two trials comparing apixaban with the European enoxaparin dose as thromboprophylaxis following elective total hip or knee replacement surgery [25,26]

	2.5 mg apixaban initiated post-surgery	40 mg enoxaparin initiated 12 h pre-surgery
**ADVANCE-2 trial (knee replacement)**		
Total VTE and all-cause mortality (primary efficacy end-point)	147/976 (15.1%)	243/997 (24.4%)
Major bleeding	9/1501 (0.6%)	14/1508 (0.9%)
**ADVANCE-3 trial (hip replacement)**		
Total VTE and all-cause mortality (primary efficacy end-point)	27/1949 (1.4%)	74/1917 (3.9%)
Major bleeding	22/2673 (0.8%)	18/2659 (0.7%)

*VTE *venous thromboembolism

## Implications

Studies comparing pre- and postoperative initiation of thromboprophylaxis show no advantage of preoperative over postoperative initiation. The historic experience together with the evidence gathered in the development of the novel oral anticoagulants dabigatran etexilate, rivaroxaban and apixaban has confirmed that postoperatively administered thromboprophylaxis is an efficacious and safe regimen.

Postoperative initiation of thromboprophylaxis with dabigatran etexilate, rivaroxaban or apixaban offers several benefits, including flexibility with regard to same-day admission and choice of anesthesia. On a practical level, because the actual time at which an operation may be initiated is uncertain (e.g. affected by preparation time, delays from previous surgical cases), it may be difficult to ensure that a dose given preoperatively provides adequate coverage during the operation itself. Additionally, administration 12 h prior to an operation may require waking patients from their sleep, which they may find disturbing and prevent them from resting before the operation.

A frequently asked question is whether or not a patient is adequately anticoagulated if they 'lose' the first oral dose due to postoperative vomiting. Analyses of pooled data from the phase III trials of dabigatran etexilate showed no significant difference in efficacy between patients who received the first dose (as a half-dose) 1-4 h post-surgery compared with those who received a delayed first dose (either a half-dose > 4 h post-surgery or the full dose the day after surgery) [55].

## Conclusion

In summary, the direct thrombin or factor Xa inhibitors dabigatran etexilate, rivaroxaban and apixaban, administered postoperatively, are at least as effective as preoperative enoxaparin and have a similar risk of major bleeding to enoxparin. The availability of these new, oral, postoperatively administered agents is likely to challenge the current practice of preoperative initiation of anticoagulant therapy in many European countries.

## Competing interests

The author declares that he repeatedly has given paid invited lectures for Boehringer Ingelheim (BI).
